# Optimal timing for surgical reconstruction of bile duct injury: meta‐analysis

**DOI:** 10.1002/bjs5.50321

**Published:** 2020-08-27

**Authors:** A. M. Schreuder, B. C. Nunez Vas, K. A. C. Booij, S. van Dieren, M. G. Besselink, O. R. Busch, T. M. van Gulik

**Affiliations:** ^1^ Department of Surgery Amsterdam UMC, University of Amsterdam Amsterdam the Netherlands; ^2^ Department of Plastic and Reconstructive Surgery Spaarne Gasthuis Haarlem the Netherlands

## Abstract

**Background:**

Major bile duct injury (BDI) after cholecystectomy generally requires surgical reconstruction by means of hepaticojejunostomy. However, there is controversy regarding the optimal timing of surgical reconstruction.

**Methods:**

A systematic review was performed by searching PubMed, Embase and Cochrane databases for studies published between 1990 and 2018 reporting on the timing of hepaticojejunostomy for BDI (PROSPERO registration CRD42018106611). The main outcomes were postoperative morbidity, postoperative mortality and anastomotic stricture. When individual patient data were available, time intervals of these studies were attuned to render these comparable with other studies. Data for comparable time intervals were pooled using a random‐effects model. In addition, data for all included studies were pooled using a generalized linear model.

**Results:**

Some 21 studies were included, representing 2484 patients. In these studies, 15 different time intervals were used. Eight studies used the time intervals of less than 14 days (early), 14 days to 6 weeks (intermediate) and more than 6 weeks (delayed). Meta‐analysis revealed a higher risk of postoperative morbidity in the intermediate interval (early *versus* intermediate: risk ratio (RR) 0·73, 95 per cent c.i. 0·54 to 0·98; intermediate *versus* delayed: RR 1·50, 1·16 to 1·93). Stricture rate was lowest in the delayed interval group (intermediate *versus* delayed: RR 1·53, 1·07 to 2·20). Postoperative mortality did not differ within time intervals. The additional analysis demonstrated increased odds of postoperative morbidity for reconstruction between 2 and 6 weeks, and decreased odds of anastomotic stricture for delayed reconstruction.

**Conclusion:**

This meta‐analysis found that surgical reconstruction of BDI between 2 and 6 weeks should be avoided as this was associated with higher risk of postoperative morbidity and hepaticojejunostomy stricture.

## Graphical abstract

This meta‐analysis addressed the timing of surgical reconstruction with hepaticojejunostomy for bile duct injury. Reconstruction between 2 and 6 weeks after injury was associated with an increased risk of postoperative morbidity as well as anastomotic stricture formation. Both early (less than 2 weeks) and delayed (more than 6 weeks) reconstruction showed lower postoperative morbidity, as well as a lower risk of anastomotic stricture.

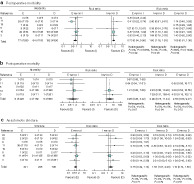

Avoid repair between 2 and 6 weeks from injury

## Abstract (es)

**Antecedentes:**

La lesión mayor de la vía biliar (*bile duct injury*, BDI) después de la colecistectomía generalmente requiere reconstrucción quirúrgica mediante una hepaticoyeyunostomía. Sin embargo, existe controversia en relación al momento óptimo para la reconstrucción quirúrgica.

**Métodos:**

Se realizó una revisión sistemática mediante la búsqueda en las bases de datos de Pubmed, Embase y Cochrane de los estudios que proporcionaban información sobre el momento de la realización de la hepaticoyeyunostomía por la BDI, publicados entre 1990‐2018 (registro PROSPERO: CRD42018106611). Los resultados principales fueron la morbilidad postoperatoria, la mortalidad postoperatoria y las estenosis de la anastomosis. Cuando se disponía de datos individuales de los pacientes, los intervalos de tiempo de estos estudios se ajustaron para hacerlos comparables con otros estudios. Los datos de los intervalos de tiempo comparables se agruparon utilizando un modelo de efectos aleatorios. Además, los datos de todos los estudios incluidos se agruparon mediante un modelo lineal generalizado.

**Resultados:**

Se incluyeron 21 estudios con un total de 2.229 pacientes. En estos estudios, se utilizaron 15 intervalos de tiempo diferentes. Ocho estudios utilizaron los intervalos de tiempo < 14 días (precoz), 14 días‐6 semanas (intermedio) y > 6 semanas (tardío). El metaanálisis reveló un mayor riesgo de morbilidad postoperatoria en el intervalo intermedio (precoz *versus* intermedio: riesgo relativo, RR 0,73; i.c. del 95%: 0,54‐0,98; intermedio *versus* tardío RR 1,50, 1,16‐1,93). La tasa de estenosis fue más baja en el grupo del intervalo tardío (RR intermedio *versus* tardío, RR 1,50, 1,07‐2,20). La mortalidad postoperatoria no difirió entre los distintos intervalos de tiempo. El análisis adicional demostró mayores probabilidades de morbilidad postoperatoria para la reconstrucción entre 2‐6 semanas y menores probabilidades de estenosis anastomóticas en el caso de reconstrucción tardía.

**Conclusión:**

Este metaanálisis muestra que la reconstrucción quirúrgica de la BDI entre 2 y 6 semanas debe evitarse, ya que este intervalo se asoció con un mayor riesgo de morbilidad postoperatoria y estenosis de la hepaticoyeyunostomía.

## Introduction

Bile duct injury (BDI) is a devastating complication following laparoscopic or open cholecystectomy. Treatment of BDI is typically tailored to the individual patient, based on the severity and type of injury and the moment of diagnosis. Minor injuries may be treated endoscopically or percutaneously, but major injuries (complete transection or even excision of a major bile duct) generally require surgical reconstruction with a Roux‐en‐Y hepaticojejunostomy (HJ)^1^, ^2^. This may entail an extensive procedure associated with a postoperative morbidity rate of 20–31 per cent, and mortality rates of 0·7–1·6 per cent^3^, ^4^. After successful recovery from surgery, patients are still at risk of long‐term complications: approximately 10–20 per cent of patients develop an anastomotic stricture, requiring repeated percutaneous dilatation or even reoperation^2^.

One factor that may influence both short‐ and long‐term outcomes of surgical reconstruction is the timing of surgical reconstruction. Delaying surgical reconstruction allows for optimization of the clinical condition of the patient as adequate sepsis control is achieved. In this period, percutaneous drainage of biloma and diversion of bile is necessary to stop intra‐abdominal leakage and to treat intra‐abdominal sepsis. Immediate or early reconstruction, however, may reduce the burden for the patient and may prevent a decline in the clinical condition in the first place. Early reconstruction may also lead to shorter duration of hospital stay and thus reduce costs^5^. Bile duct ischaemia, however, may still be developing at the time of an early repair, eventually causing strictures proximal to the level of the anastomosis. This is especially the case when there is concomitant vascular injury.

There appears to be no systematic review on this topic, whereas debate on the optimal timing of HJ for BDI has continued in the past decade. Studies on this topic report conflicting results, using a wide variation of cut‐off times for ‘early’ and ‘delayed’ surgery. The aim of this systematic review and meta‐analysis was to assess the timing of surgical reconstruction with HJ as a risk factor for postoperative morbidity, mortality, and long‐term complications in patients with major BDI following cholecystectomy.

## Methods

This systematic review was performed in accordance with the PRISMA statement^6^ and the Meta‐analysis of Observational Studies in Epidemiology (MOOSE) guideline^7^. The review protocol was registered in PROSPERO under registration number CRD42018106611.

### Eligibility criteria

All studies reporting on the effect of timing of surgical reconstruction of BDI following cholecystectomy by means of a Roux‐en‐Y HJ were intended to be included. Eligible studies had to report on one or more of the following predefined outcomes: overall postoperative (short‐term) morbidity, postoperative (short‐term) mortality, incidence of anastomotic stricture (long‐term), incidence of HJ leakage, recurrent cholangitis, and long‐term mortality.

Expecting no randomized trials on this topic, both prospective and retrospective cohorts were included. Case reports or cohorts with fewer than ten patients and review articles were excluded. Studies in which various reconstructive surgical procedures were performed (including primary repair or end‐to‐end anastomosis of the bile duct) without separately reporting patients who underwent HJ were also excluded. When patients undergoing HJ were reported separately from patients having other surgical procedures, the data for patients who had HJ were included. To avoid double‐counting patients, only the largest and most recent publication was included when two studies had overlapping patient cohorts. If patient cohorts overlapped but different outcome measures were reported, both studies were included.

### Search strategy and selection of studies

To identify relevant articles, a literature search was performed of PubMed, Embase and the Cochrane Library databases. A clinical librarian was consulted on the search strategy. The following keywords were used: bile duct injury, cholecystectomy, surgical repair, surgery, hepaticojejunostomy, biliodigestive anastomosis, timing, time, immediate, early, intermediate, late, delayed. The full search string is available in *Appendix* *S1* (supporting information). The search was limited to dates later than 1990, and the last update of the search was performed on 10 August 2018. No language restriction was applied. In addition, reference lists of all relevant articles were hand‐searched for any other eligible articles. If full texts could not be retrieved, or if reported outcomes were incomplete, the corresponding authors were contacted by e‐mail.

Titles and abstracts were screened independently by two authors using Rayyan online software (https://rayyan.qcri.org/)^8^. Full texts of articles were obtained if they matched the eligibility criteria or if further scrutiny was needed regarding eligibility. Any disagreements about eligibility were resolved by consensus or by the senior author.

### Quality assessment

Two authors critically appraised all studies using the Newcastle–Ottawa Scale (NOS). This scale is a validated scoring system for quality assessment of observational cohort studies in systematic reviews, and leads to a score of up to 9 points. Any disagreements were resolved by consensus.

### Data collection

Data collection was done by two authors using predesigned spreadsheets. Collected data included: study characteristics (author, publication year, study design, number of patients, inclusion period, follow‐up), time frames used, and outcomes per time frame. Primary outcomes included anastomotic stricture, overall morbidity and postoperative mortality. Secondary outcomes included length of stay, bile leakage, haemorrhage, intra‐abdominal abscess, hepatolithiasis, long‐term BDI‐related mortality, reintervention and re‐repair.

All corresponding authors of the included studies were contacted by e‐mail and asked to share individual patient data for these studies. Per patient, information on the primary outcomes as well as the number of days between injury and HJ was requested. These data were used to attune time frames in order to be able to compare studies.

### Statistical analysis

For meta‐analysis on the primary outcomes, studies were considered comparable if they used the same time frames for early, intermediate and delayed reconstruction. For studies where individual patient data were provided by the authors, outcomes were recalculated according to the time frame most used across studies. Meta‐analyses were performed by inverse‐variance weighting with a random‐effects model using the meta package (version 4.9‐2) for R software (version 3.4.3) (R Foundation for Statistical Computing, Vienna, Austria). Heterogeneity was assessed using the χ^2^ test and the *I* ^2^ statistic. An *I* ^2^ value of 30–50 per cent was considered to represent moderate heterogeneity and 50 per cent or above was considered to represent substantial heterogeneity. Results were visualized in forest plots.

To utilize all available data reported by the included studies (irrespective of the time frames chosen by authors), a generalized linear model was used to estimate mean odds for the primary outcomes per time interval (day 0–7, week 2–26). This analysis was done using SPSS® version 24.0.0.1 (IBM, Armonk, New York, USA). For this, odds were calculated per time interval used by a study. These odds were converted to odds per day and weighted for the number of patients per day (calculated by dividing the number of patients in this time interval by the number of days in the interval). For time intervals containing no events, a correction factor of half an event was applied to calculate the odds. Subsequently, a generalized linear model was used to calculate estimated mean odds per time interval. The odds per time interval was the dependent variable in this model, with the study and the time interval as (nominal) independent variables. The resulting estimated mean odds and 95 per cent c.i. were visualized in graphical form. Owing to the low number of events, this analysis was omitted for the primary outcome mortality.

## Results

**Fig. 1:**
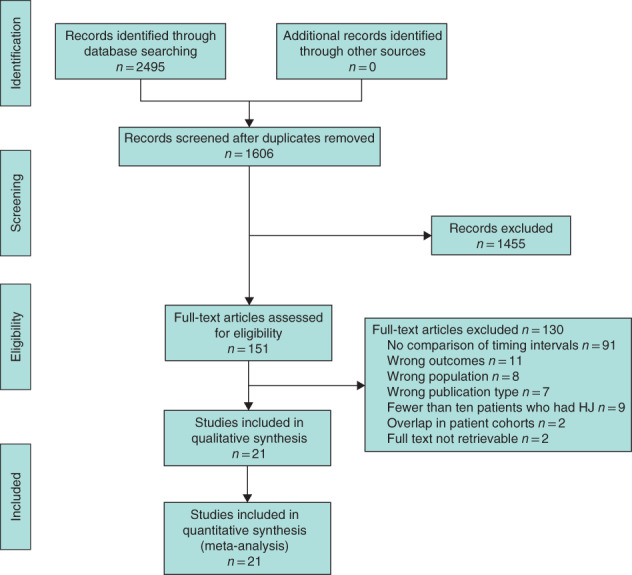
PRISMA diagram for the review HJ, hepaticojejunostomy.

The search identified 2495 records (*Fig*. *1*). After removal of duplicates, 1606 unique records remained. A total of 130 studies were excluded based on the full text screening; reasons for exclusion per article are listed in *Table S1* (supporting information). In total, 21 studies^3^, ^4^, ^5^, ^9^, ^10^, ^11^, ^12^, ^13^, ^14^, ^15^, ^16^, ^17^, ^18^, ^19^, ^20^, ^21^, ^22^, ^23^, ^24^, ^25^, ^26^ were included in the review and meta‐analysis, representing a total of 2484 patients. For five studies, individual patient data were extracted from the article^9^ or provided by the authors^3^, ^5^, ^10^, ^11^.

### Study characteristics and critical appraisal

All included studies were observational and retrospective, although five studies^3^, ^10^, ^12^, ^13^, ^14^ (comprising 1045 patients) used a prospectively maintained database (*Table* *1*). Fourteen of the 21 studies were published in or after 2010.

**Table 1:** Study characteristics

**Reference**	**Study design**	**Setting**	**Inclusion**	**HJ (*n*)**	**Technique of HJ**	**Short‐term outcomes**	**Long‐term outcomes**	**Follow‐up (months)** *	**NOS score**
Ooi *et al*.^24^	R	Singapore, 4 teaching hospitals	1990–1997	14	n.s.	–	Stricture, cholangitis, mortality	51 (8–83)	5
Thomson *et al*.^14^	R†	UK, single HPB centre	1988–2003	47	At level of biliary confluence	Mortality, reintervention	–	33 (6–201)	6
Akaraviputh *et al*.^12^	R†	Thailand, single university hospital	1992–2005	19	E‐S, diameter ≥ 2 cm	Morbidity, mortality, LOS, reintervention	Cholangitis	22 (1–120)	5
Walsh *et al*.^25^	R	USA, single HPB centre	1990–2005	84	Single‐layer interrupted sutures; stents used selectively	Mortality	Stricture	97 (21–175)	8
Goykhman *et al*.^15^	R	Israel, single HPB centre	2002–2007	23	Single‐layer interrupted sutures; stents used selectively	Bile leak	Stricture	24 (12–60)	6
Stewart and Way^23^	R	USA, single HPB centre	–	137	E‐S, single‐layer	–	Stricture	40	7
Winslow *et al*.^21^	R	USA, single HPB centre	1992–2006	88	S‐S	Morbidity, mortality	Stricture, re‐repair	4·3 years	7
Sahajpal *et al*.^16^	R	Canada, 2 teaching hospitals	1992–2007	69	n.s.	Morbidity, mortality	Stricture	71·5 (0–120)	7
Perera *et al*.^13^	R†	UK, single HPB centre	1991–2007	112	n.s.	–	Stricture, cholangitis, re‐repair, morbidity	55 (0–186)	6
Iannelli *et al*.^4^	R	France, 47 hospitals	1990–2010	253	n.s.	Morbidity, mortality	Reintervention	n.r.	5
Pitt *et al*.^22^	R	USA, single HPB centre	1993–2010	101	With stent	–	Stricture	Mean 4·1 years	6
Gluszek *et al*.^9^	R	Poland, single centre	–	11	n.s.	Morbidity, mortality	Stricture, cholangitis	n.r.	5
Huang *et al*.^20^	R	China, single centre	1998–2010	94	n.s.	Morbidity	Stricture	65·5 (6–120)	6
Stilling *et al*.^11^	R	Denmark, 5 HPB centres	1995–2010	139	E‐S, no stent	Morbidity, mortality, HJ leakage	Stricture, cholangitis	114 (0–182)	6
Felekouras *et al*.^19^	R	Greece, single HPB centre	1991–2011	56	E‐S, with stent	Morbidity, bile leak	Stricture, cholangitis, re‐repair, mortality	7·75 years (8–230 months)	7
Gomes and Doctor^17^	R	India, single HPB centre	1999–2011	40	Hepp–Couinaud	Morbidity	–	7 years (36–120 months)	8
Rystedt *et al*.^5^	R	Sweden, national registry (76 hospitals)	2007–2011	30	n.s.	LOS	Stricture	3 years	6
Dominguez‐Rosado *et al*.^10^	R†	Mexico, single centre	1989–2014	586	S‐S, Hepp–Couinaud	Morbidity	–	22 (1–258)	7
Kirks *et al*.^26^	R	USA, single HPB centre	2008–2015	61	n.s.	Mortality, total LOS	–	n.r.	7
Ismael *et al*.^18^	R	USA, NSQIP registry	2005–2012	239	n.s.	Morbidity, mortality	–	30 days	6
Booij *et al*.^3^	R†	Netherlands, single HPB centre	1991–2016	281	According to Couinaud; stents used selectively	Morbidity, mortality, bile leak, reintervention	Stricture	10·5 (i.q.r. 6·7–14·8) years	7

* Values are median (range) months unless indicated otherwise.

†Retrospective (R) analysis of prospectively maintained database. HJ, hepaticojejunostomy; NOS, Newcastle–Ottawa Scale; n.s., not specified; HPB, hepatopancreatobiliary; NSQIP, National Surgical Quality Improvement Program; E‐S, end‐to‐side; LOS, length of stay; S‐S, side‐to‐side; n.r., not reported.

NOS risk‐of‐bias scores varied between 5 and 8 (*Table* *1*; *Table S2*, supporting information). Most studies did not report on completeness of follow‐up, and median follow‐up was less than 5 years or not reported in 14 studies. As this review included only patients who underwent HJ for BDI, treatment exposure was ascertained in all studies.

Across the 21 included studies, 12 different time points were used to define time intervals, in different arrangements. This resulted in 15 different sets of time frame used across studies (*Table S3*, supporting information). The most commonly used time frame was less than 14 days for early reconstruction, more than 14 days to 6 weeks for intermediate reconstruction, and more than 6 weeks for delayed reconstruction. After attuning data for the studies that provided individual patient data to these intervals, eight studies could be included in a meta‐analysis for the outcome anastomotic stricture, six studies for the outcome postoperative morbidity, and five studies for the outcome postoperative mortality.

### Unpooled results

**Fig. 2:**
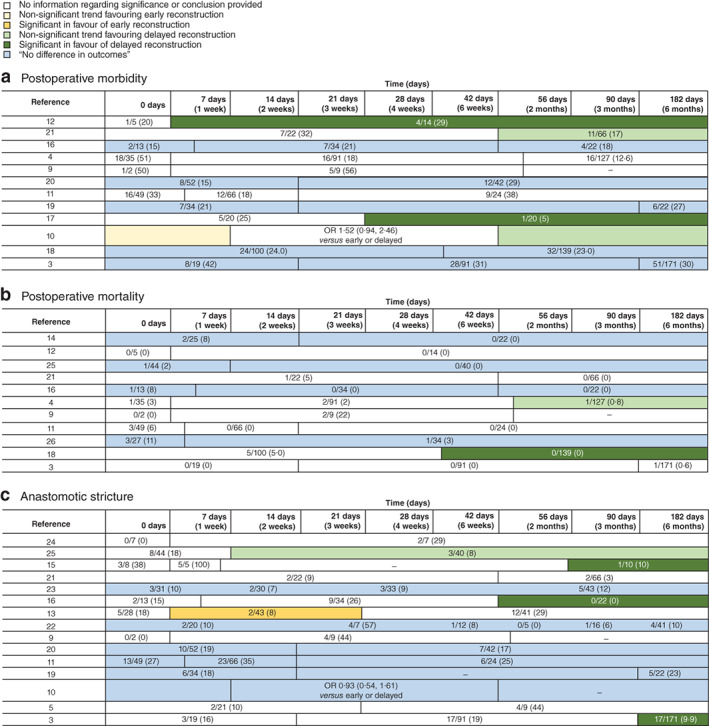
Data for primary outcomes according to time intervals, as provided by the studies **a** Postoperative morbidity; **b** postoperative mortality; **c** anastomotic stricture. Values in parentheses are percentages. OR, odds ratio. The key indicates the conclusion as provided by the studies.

Primary outcomes as reported by the original studies are presented in *Fig*. *2* and secondary outcomes in *Fig. S1* (supporting information). Most studies found no significant differences between timing intervals for any of the primary outcomes, or did not mention statistical significance. In 15 studies, anastomotic stricture was a reported outcome measure, with reported stricture rates ranging from 5 to 39 per cent. Of these, three studies^3^, ^15^, ^16^ found significantly lower rates of stricture in patients undergoing delayed reconstruction, and one study^13^ found a significantly lower stricture rate in patients having reconstruction between 1 and 3 weeks.

Postoperative morbidity was reported in 12 studies, with morbidity rates varying between 15 and 54 per cent. Two studies^4^, ^17^ found a significantly lower rate of morbidity in patients who had a delayed reconstruction. Of 11 studies reporting postoperative mortality, one study^18^ reported a significantly lower rate in patients undergoing delayed reconstruction. Reported mortality rates ranged from 0 to 18 per cent, the latter in a small study^9^ with only 11 patients.

### Pooled results for postoperative morbidity

**Fig. 3:**
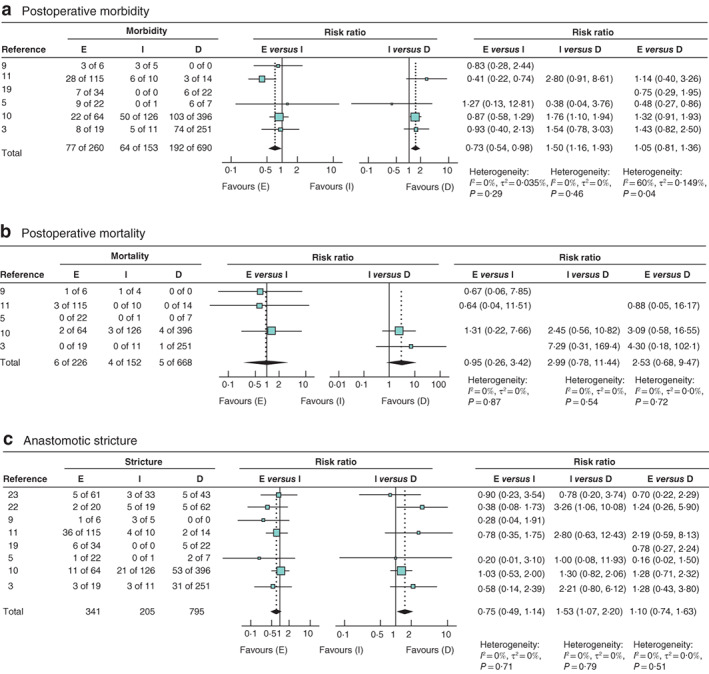
Forest plots comparing risk ratios for early (less than 14 days), intermediate (14–42 days) and delayed (more than 42 days) reconstruction of bile duct injury **a** Postoperative morbidity; **b** postoperative mortality; **c** anastomotic stricture. An inverse‐variance random‐effects model was used for meta‐analysis. Risk ratios are shown with 95 per cent confidence intervals. E, early reconstruction; I, intermediate reconstruction; D, delayed reconstruction.

Six studies, comprising 1103 patients, were included in the meta‐analysis of postoperative morbidity (*Fig*. *3a*). Early reconstruction (less than 14 days) was associated with a lower risk of morbidity compared with intermediate reconstruction (15 days to 6 weeks) (risk ratio (RR) 0·73, 95 per cent c.i. 0·54 to 0·98), as was delayed reconstruction (more than 6 weeks) (intermediate *versus* delayed: RR 1·50, 1·16 to 1·93). Heterogeneity was low for both comparisons (*I* ^*2*^ = 0 per cent). No significant difference was seen between early and delayed reconstruction (RR 1·05, 0·81 to 1·36), and heterogeneity for this comparison was high (*I* ^*2*^ = 60 per cent).

**Fig. 4:**
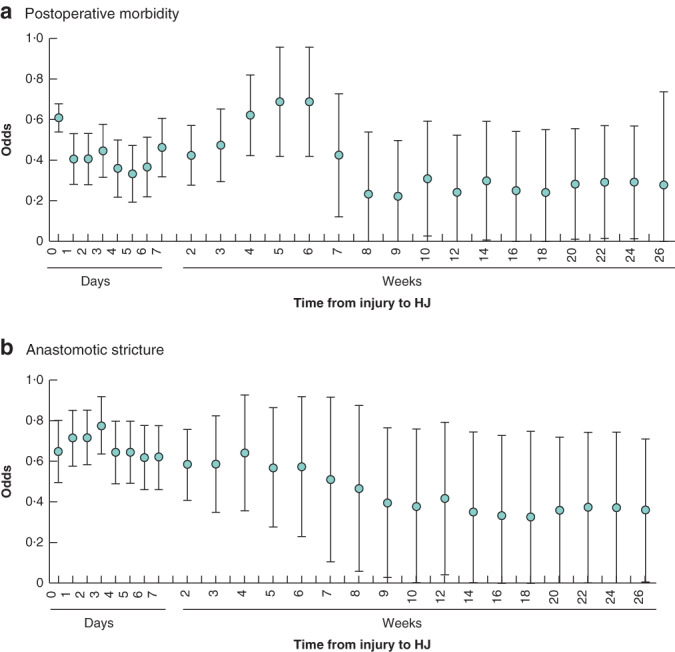
Estimated odds for the association between time from injury to repair and postoperative morbidity and anastomotic stricture **a** Postoperative morbidity; **b** anastomotic stricture. Mean odds with 95 per cent c.i. values are shown. HJ, hepaticojejunostomy.

Data for all 12 studies^3^, ^4^, ^9^, ^10^, ^11^, ^12^, ^16^, ^17^, ^18^, ^19^, ^20^, ^21^ reporting on postoperative morbidity (comprising 1875 patients) were entered into the generalized linear model. The resulting estimated mean odds and corresponding c.i. are presented in *Fig*. *4a*. Estimated mean odds for morbidity were increased between 3 and 6 weeks.

### Pooled results for postoperative mortality

Five studies, comprising 1046 patients, were included in the meta‐analysis of postoperative mortality (*Fig*. *3b*). There was no significant association between timing of repair and postoperative mortality.

Owing to the low number of events, no conversion to odds and subsequent generalized linear model was performed for this outcome.

### Pooled results for anastomotic stricture

Eight studies^3^, ^5^, ^9^, ^10^, ^11^, ^19^, ^22^, ^23^, comprising 1341 patients, were included in the meta‐analysis of anastomotic stricture (*Fig*. *3c*). For early *versus* intermediate reconstruction, no significant difference was found in stricture rate (RR 0·75, 95 per cent c.i. 0·49 to 1·14). Comparing intermediate with delayed reconstruction, intermediate reconstruction was associated with a higher risk of anastomotic stricture (RR 1·53, 1·07 to 2·20). There was no significant difference in risk of anastomotic stricture for early *versus* delayed reconstruction (RR 1·10, 0·74 to 1·63). For all three of these meta‐analyses, heterogeneity was low (*I* ^*2*^ = 0 per cent).

Data for all 15 studies^3^, ^5^, ^9^, ^10^, ^11^, ^13^, ^15^, ^16^, ^19^, ^20^, ^21^, ^22^, ^23^, ^24^, ^25^ reporting on anastomotic stricture (comprising 1821 patients) were entered into the generalized linear model. The resulting estimated mean odds and corresponding confidence intervals are presented in *Fig*. *4b*. Mean odds gradually decreased with a longer time interval between injury and reconstruction; lowest mean odds appear to be from week 9 after the injury and subsequently.

## Discussion

Timing of surgical reconstruction for major BDI has been a continuing topic of debate. This systematic review and meta‐analysis demonstrates that delaying surgical reconstruction for at least 6 weeks is associated with lower postoperative morbidity rates and lower risk of anastomotic stricture compared with intermediate reconstruction (2–6 weeks). Early reconstruction (within 2 weeks) was also associated with a lower risk of postoperative morbidity than intermediate reconstruction, but may pose a slightly higher risk of anastomotic stricture. Based on these data, reconstruction in the intermediate interval (2–6 weeks) should be avoided.

Although there are several international guidelines^27^, ^28^, ^29^ providing recommendations on how to avoid BDI during cholecystectomy, they generally do not elaborate on the treatment of BDI. As this is the first systematic review on the timing of reconstruction in patients with major BDI, no comparison can be made with other (earlier) reviews. A recently published collaborative retrospective study from the European–African HepatoPancreatoBiliary Association^30^, which could not be included in this systematic review because of overlapping patient cohorts, concluded that ‘the timing of biliary reconstruction with hepaticojejunostomy did not have any impact on severe postoperative complications, the need for re‐intervention or liver‐related mortality’. However, in multivariable analysis, there was a trend towards a higher morbidity rate following early reconstruction (in this study^30^ defined as less than 7 days), nearly reaching significance (reconstruction later than 42 days *versus* early reconstruction: odds ratio 0·66, 95 per cent c.i. 0·43 to 1·02, *P* = 0·058). The study design is prone to selection bias as local policies of participating centres may have influenced its outcome. Furthermore, the follow‐up period in this study was only 2 years, which is relatively short for detection of anastomotic stricture. Still, this large study^30^ of 913 patients with major BDI reported contradicting results compared with the present meta‐analysis, indicating that there is still a need for better evidence on this topic, for example from a prospective registry. Such a registry would require a uniform classification of the severity of BDI, as well as predefined outcomes and registration of possible confounders such as sepsis and vascular injury.

Several studies^13^, ^15^, ^19^, ^23^ have demonstrated significantly better outcomes following repair of BDI when performed by a specialized hepatopancreatobiliary (HPB) surgeon compared with general surgeons. Besides experience in hepatobiliary surgery, these differences may in part be explained by the surgical technique used: HPB surgeons tended to use HJ, whereas general surgeons performed an end‐to‐end anastomosis in the majority of patients. This technique is considered inferior to HJ because of the extremely high rates of reported stricture formation^31^. Another explanation for differences in outcomes between HPB and general surgeons may be a less than optimal workup by general surgeons. For example, in the study by Stewart and Way^23^ only 36 per cent of patients treated by a general surgeon had adequate drainage of biloma before reconstruction, leading to insufficient control of intra‐abdominal inflammation. This emphasizes the importance of early referral to a centre with expertise in BDI, even when surgical reconstruction is delayed and initially only drainage is performed^32^, ^33^.

The results of this systematic review should be interpreted in the light of several limitations. First, only observational cohort studies could be included, inevitably leading to selection bias. The time of detection of BDI may be the most important source of selection bias: only about 20–40 per cent of BDIs are recognized during the initial cholecystectomy^3^, ^11^, ^19^. Patients in whom the injury is detected weeks after cholecystectomy can never undergo early reconstruction. It is, however, rare for a major BDI to be detected so late. Second, most study results were not adjusted for possible confounders, such as vascular injury or sepsis. A meta‐analysis with adjusted study results was not possible owing to the lack of relevant data. Patients with a worse clinical condition in the presence of sepsis or biliary peritonitis may have been more likely to have reconstruction in the delayed phase, after waiting for the inflammatory sequelae to subside. It is therefore likely that there is a substantial degree of confounding by indication in this meta‐analysis. This group may also contain more patients with a failure of primary repair, which has been reported to be associated with a worse outcome of reconstruction^34^. However, as the delayed group is expected to consist of ‘worse’ patients, the confounding by indication probably does not attenuate the present conclusion. Local hospital policies may also have played a role in treatment allocation, although series reporting on only early reconstruction or only delayed reconstruction were excluded from this review. Third, the time intervals used in this review were dependent on the intervals as defined in the original studies, which were often based on local preference. Because of the many different time intervals (15), pooling of data was initially not possible. An effort was made to include as many reliable data as possible in the meta‐analysis, by reaching out to the individual authors for individual patient data. In addition, by applying two different statistical approaches, the strength of the study was enhanced whilst, despite some differences in study characteristics, statistical heterogeneity was low in all analyses.

The conclusions drawn from this study are based on the outcomes for anastomotic stricture, postoperative morbidity and postoperative mortality. These outcomes were reported by most of the included studies, and can be considered most clinically relevant. Postoperative morbidity and mortality obviously affect patients in the postoperative period. Anastomotic stricture requires repeated percutaneous balloon dilatation, often for a period of more than 3 months^35^, or ultimately surgical reintervention. The invasive nature of these treatments contributes significantly to the long‐term impact of BDI and the impaired quality of life experienced by patients even years after sustaining BDI^2^, ^36^. When left untreated, anastomotic stricture with recurrent cholangitis may cause secondary biliary cirrhosis and end‐stage liver disease.

One outcome less investigated, but also of importance, is healthcare costs. It seems plausible that patients undergoing delayed reconstruction need more frequent procedures and longer total in‐hospital stay, as preoperative optimization may require repeated admissions. Although some studies have demonstrated a shorter total hospital stay^5^ and lower costs^37^ in patients undergoing early repair of BDI, they did not take into account the long‐term costs of reinterventions for anastomotic stricture. Achieving optimal long‐term outcomes remains most important in treatment of BDI.

## Collaborators

N. Stilling (Department of Surgery, Odense University Hospital, Odense, Denmark); I. Dominguez Rosado and M. Mercado (Department of Surgery, National Institute of Medical Science and Nutrition ‘Salvador Zubiran’, Mexico City, Mexico); J. Rystedt (Department of Surgery, Skåne University Hospital; Lund University, Lund, Sweden).

## Supporting information


**Appendix S1:** Supporting information
