# Supplementary material for: Optimal timing for surgical reconstruction of bile duct injury: meta‐analysis
Source: BJS Open. 2020 Aug 27;4(5):776–86. doi: 10.1002/bjs5.50321 (PMC7528508; doi:10.1002/bjs5.50321)
Supplement: Supplementary file 1 — Appendix S1: Supporting information [file BJS5-4-776-s001.docx]

**BJS5_50231**

**Optimal timing for surgical reconstruction of iatrogenic bile duct injury: meta-analysis**

**A. M. Schreuder, B. C. Nunez Vas, K. A. C. Booij, S. van Dieren, M. G. Besselink, O. R. Busch and T. M. van Gulik**

**Appendix S1** Search strategy

**PUBMED**

("Bile Ducts/injuries"[Mesh] OR bile duct injur*[tiab] OR biliary injur*[tiab] OR bile leak*[tiab] OR bile duct strictur*[tiab]) AND ("Biliary Tract Surgical Procedures"[Mesh:NoExp] OR "Cholecystectomy"[Mesh] OR cholecystectom*[tiab]) AND ("Surgical Procedures, Operative"[Mesh] OR "surgery" [Subheading] OR "Choledochostomy"[Mesh] OR surgical repair*[tiab] OR surgical reconstruction*[tiab] OR biliary reconstruction*[tiab] OR surgery[tiab] OR surgeries[tiab] OR hepaticojejunostom*[tiab] OR choledochoduodenostom*[tiab] OR hepaticoduodenostom*[tiab] OR biliodigestive anastomos*[tiab]) AND ("Time Factors"[Mesh] OR "Time-to-Treatment"[Mesh] OR early[tiab] OR earlier[tiab] OR late[tiab] OR time[tiab] OR timing[tiab] OR delay*[tiab] OR immediat*[tiab]) NOT ("Case Reports" [Publication Type] OR case report*[tiab]) AND (english[Language] OR dutch[Language]) AND ("2018/03/01"[Date - Publication] : "3000"[Date - Publication])

**EMBASE (Ovid):**

| **#** | **Searches** | **Results** |
| --- | --- | --- |
| 1 | bile duct injury/ or (bile duct injur* or biliary injur* or bile leak* or bile duct strictur*).ti,ab,kw. | 10511 |
| 2 | exp biliary tract surgery/ or exp cholecystectomy/ or cholecystectom*.ti,ab,kw. | 76367 |
| 3 | exp surgery/ or surgery.fs. or exp bile duct bypass/ or (surgical repair* or surgical reconstruction* or biliary reconstruction* or surgery or surgeries or hepaticojejunostom* or choledochoduodenostom* or hepaticoduodenostom* or biliodigestive anastomos*).ti,ab,kw. | 5149930 |
| 4 | time factor/ or time to treatment/ or (early or earlier or late or time or timing or delay* or immediat*).ti,ab,kw. | 6244337 |
| 5 | 1 and 2 and 3 and 4 | 2444 |
| 6 | limit 5 to conference abstract status | 884 |
| 7 | 5 not 6 | 1560 |
| 8 | case report/ or case report*.ti,ab,kw. | 2331106 |
| 9 | 7 not 8 | 1400 |

**Cochrane Library**


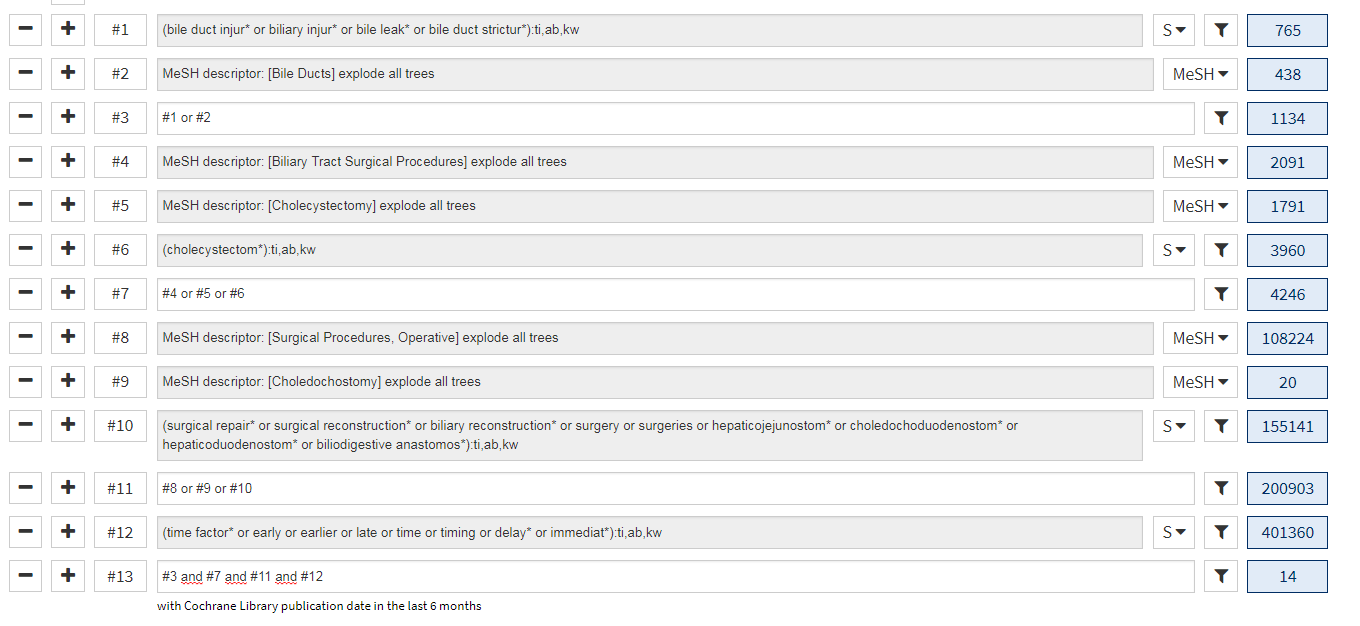


**Table S1 Articles excluded after full-text assessment for eligibility**

| Reference | Reason for exclusion |
| --- | --- |
| Abdel Wahab^1^ 1996 | No comparison of outcomes based on timing of repair |
| AbdelRafee^2^ 2015 | No comparison of outcomes based on timing of repair |
| Adams^3^ 1992 | Less than 10 hepaticojejunostomies |
| Ali^4^ 2014 | No comparison of outcomes based on timing of repair |
| Andrén-Sandberg^5^ 1985 | No comparison of outcomes based on timing of repair |
| Asbun^6^ 1993 | No comparison of outcomes based on timing of repair |
| Bachellier^7^ 2001 | No comparison of outcomes based on timing of repair |
| Bakhsh^8^ 2002 | Wrong study population |
| Bansal^9^ 2013 | No comparison of outcomes based on timing of repair |
| Barros^10^ 2010 | No comparison of outcomes based on timing of repair |
| Barrow^11^ 2007 | No comparison of outcomes based on timing of repair |
| Bauer^12^ 1998 | No comparison of outcomes based on timing of repair |
| Bektas^13^ 2007 | No comparison of outcomes based on timing of repair |
| Bingham^14^ 2000 | No comparison of outcomes based on timing of repair |
| Bobkiewicz^15^ 2014 | No comparison of outcomes based on timing of repair |
| Boerma^16^ 2001 | No comparison of outcomes based on timing of repair |
| Branum^17^ 1993 | No comparison of outcomes based on timing of repair |
| Browder^18^ 1986 | No comparison of outcomes based on timing of repair |
| Buell^19^ 2002 | No comparison of outcomes based on timing of repair |
| Cai^20^ 2014 | No comparison of outcomes based on timing of repair |
| Chaudhary^21^ 2002 | Wrong study population |
| Cieslicki^22^ 1997 | Wrong article type |
| Csendes^23^ 1989 | Less than 10 hepaticojejunostomies |
| Cuendis-Velazquez^24^ 2018 | No comparison of outcomes based on timing of repair |
| Cuendis-Velazquez^25^ 2015 | No comparison of outcomes based on timing of repair |
| Dageforde^26^ 2012 | Wrong article type |
| de Reuver^27^ 2007 | Overlap with patient cohorts of recent publication |
| St. van de Sande^28^ 2003 | Wrong outcomes reported |
| de Santibanes^29^ 2006 | No comparison of outcomes based on timing of repair |
| Efskind^30^ 1956 | Wrong study population / before 1980 |
| Elhamel^31^ 2008 | No comparison of outcomes based on timing of repair |
| Fathy^32^ 2011 | No comparison of outcomes based on timing of repair |
| Fischer^33^ 2008 | No comparison of outcomes based on timing of repair |
| Frilling^34^ 2004 | No comparison of outcomes based on timing of repair |
| Gazzaniga^35^ 2001 | No comparison of outcomes based on timing of repair |
| Giulianotti^36^ 2017 | No comparison of outcomes based on timing of repair |
| Gouma^37^ 1994 | No comparison of outcomes based on timing of repair |
| Gouma^38^ 1999 | Wrong article type |
| Gupta^39^ 2013 | No comparison of outcomes based on timing of repair |
| Hadi^40^ 2013 | No comparison of outcomes based on timing of repair |
| Hajjar^41^ 2014 | No comparison of outcomes based on timing of repair |
| Halbert^42^ 2016 | No comparison of outcomes based on timing of repair |
| Hart^43^ 2000 | No comparison of outcomes based on timing of repair |
| Helmy^44^ 1997 | No comparison of outcomes based on timing of repair |
| Hofmeyer^45^ 2015 | Wrong outcomes reported |
| Hogan^46^ 2016 | Wrong outcomes reported |
| Holte^47^ 2010 | No comparison of outcomes based on timing of repair |
| Huang, Liuz^48^ 2013 | Wrong study population |
| Huang, Shao^49^ 2011 | Overlap with patient cohorts of recent publication |
| Jablonska^50^ 2008 | No comparison of outcomes based on timing of repair |
| Jackson^51^ 2016 | No comparison of outcomes based on timing of repair |
| Jayasundara^52^ 2011 | No comparison of outcomes based on timing of repair |
| Johnson^53^ 2000 | No comparison of outcomes based on timing of repair |
| Joshi^54^ 2008 | No comparison of outcomes based on timing of repair |
| Kaman^55^ 2006 | No comparison of outcomes based on timing of repair |
| Karabulut^56^ 2012 | Less than 10 hepaticojejunostomies |
| Karvonen^57^ 2007 | No comparison of outcomes based on timing of repair |
| Kazibudzki^58^ 2006 | No comparison of outcomes based on timing of repair |
| Kerimoglu^59^ 2017 | Wrong study population |
| Keulemans^60^ 1998 | No comparison of outcomes based on timing of repair |
| Krahnenbuhl^61^ 2001 | Wrong outcomes reported |
| Kune^62^ 1979 | No comparison of outcomes based on timing of repair |
| Li^63^ 2005 | Less than 10 hepaticojejunostomies |
| Lillemoe^64^ 1997 | No comparison of outcomes based on timing of repair |
| Lillemoe^65^ 2000 | No comparison of outcomes based on timing of repair |
| Linhares^66^ 2011 | No comparison of outcomes based on timing of repair |
| Lubikowski^67^ 2010 | No comparison of outcomes based on timing of repair |
| Ludwig^68^ 2001 | Wrong outcomes reported |
| Maddah^69^ 2015 | No comparison of outcomes based on timing of repair |
| Martinez-Lopez^70^ 2017 | No comparison of outcomes based on timing of repair |
| Mathisen^71^ 2002 | No comparison of outcomes based on timing of repair |
| Mercado^72^ 2003 | No comparison of outcomes based on timing of repair |
| Mercado^73^ 2007 | No comparison of outcomes based on timing of repair |
| Mercado^74^ 2011 | No comparison of outcomes based on timing of repair |
| Mihaileanu^75^ 2012 | No comparison of outcomes based on timing of repair |
| Mishra^76^ 2013 | No comparison of outcomes based on timing of repair |
| Moossa^77^ 1990 | No comparison of outcomes based on timing of repair |
| Moraca^78^ 2002 | No comparison of outcomes based on timing of repair |
| Nordin^79^ 2002 | Wrong outcomes reported |
| Nuzzo^80^ 2011 | Wrong article type |
| Nuzzo^81^ 2008 | No comparison of outcomes based on timing of repair |
| Ozturk^82^ 2009 | No comparison of outcomes based on timing of repair |
| Parks^83^ 1994 | No comparison of outcomes based on timing of repair |
| Patrono^84^ 2015 | No comparison of outcomes based on timing of repair |
| Pekolj^85^ 2013 | Less than 10 hepaticojejunostomies |
| Pottakkat^86^ 2006 | Wrong study population |
| Pulitan^87^ 1993 | Wrong article type |
| Quintero^88^ 2001 | Wrong study population |
| Raute^89^ 1993 | No comparison of outcomes based on timing of repair |
| Ress^90^ 1992 | No comparison of outcomes based on timing of repair |
| Richardson^91^ 1996 | Wrong outcomes reported |
| Riggs^92^ 1986 | Less than 10 hepaticojejunostomies |
| Rosenqvist^93^ 1960 | Year of publication before 1980 |
| Rossi^94^ 1992 | No comparison of outcomes based on timing of repair |
| Roy^95^ 1993 | Wrong outcomes reported |
| Russell^96^ 1996 | Wrong outcomes reported |
| Rystedt^97^ 2016 | No comparison of outcomes based on timing of repair |
| Saber^98^ 1984 | No comparison of outcomes based on timing of repair |
| Sadegh^99^ 2016 | No comparison of outcomes based on timing of repair |
| Salama^100^ 2014 | No comparison of outcomes based on timing of repair |
| Sanei^101^ 2018 | No comparison of outcomes based on timing of repair |
| Savar^102^ 2004 | No comparison of outcomes based on timing of repair |
| Sawaya^103^ 2001 | Wrong outcomes reported |
| Schmidt^104^ 2004 | No comparison of outcomes based on timing of repair |
| Schol^105^ 1995 | No comparison of outcomes based on timing of repair |
| Seeliger^106^ 2002 | No comparison of outcomes based on timing of repair |
| Shah^107^ 2000 | Wrong outcomes reported |
| Shaikh^108^ (?) | No comparison of outcomes based on timing of repair |
| Sicklick^109^ 2005 | No comparison of outcomes based on timing of repair |
| Sikora^110^ 2001 | Less than 10 hepaticojejunostomies |
| Sikora^111^ 2005 | Wrong study population |
| Slater^112^ 2002 | No comparison of outcomes based on timing of repair |
| Strasberg^113^ 2000 | No comparison of outcomes based on timing of repair |
| Sulpice^114^ 2013 | No comparison of outcomes based on timing of repair |
| Thomson^115^ 2003 | No comparison of outcomes based on timing of repair |
| Topal^116^ 1998 | No comparison of outcomes based on timing of repair |
| Tropea^117^ 2016 | No comparison of outcomes based on timing of repair |
| Tsalis^118^ 2002 | No comparison of outcomes based on timing of repair |
| Viikari^119^ 1960 | Year of publication before 1980 |
| Viste^120^ 2015 | No comparison of outcomes based on timing of repair |
| Walsh^121^ 1997 | No comparison of outcomes based on timing of repair |
| Wei^122^ 2011 | Wrong article type |
| Woods^123^ 1994 | No comparison of outcomes based on timing of repair |
| Wu^124^ 2007 | No comparison of outcomes based on timing of repair |
| Wudel^125^ 2001 | No comparison of outcomes based on timing of repair |
| Xu^126^ 2011 | No comparison of outcomes based on timing of repair |
| Yeh^127^ 1998 | No comparison of outcomes based on timing of repair |

**References**

1. Abdel Wahab M, el-Ebiedy G, Sultan A, et al. Postcholecystectomy bile duct injuries: experience with 49 cases managed by different therapeutic modalities. *Hepatogastroenterology.*43(11):1141-1147.

2. AbdelRafee A, El-Shobari M, Askar W, Sultan AM, El Nakeeb A. Long-term follow-up of 120 patients after hepaticojejunostomy for treatment of post-cholecystectomy bile duct injuries: A retrospective cohort study. *Int J Surg.*18:205-210.

3. Adams DB, Borowicz MR, Wootton FT, 3rd, Cunningham JT. Bile duct complications after laparoscopic cholecystectomy. *Surg Endosc.*7(2):79-83.

4. Ali SAS, Sheikh BA, Idrees F, et al. Management of iatrogenic bile duct injuries following cholecystectomy. *Medical Forum Monthly.*28(1):14-17.

5. S. A-, berg A, Johansson S, Bengmark S. Accidental lesions of the common bile duct at cholecystectomy. II. Results of treatment. *Ann Surg.*201(4):452-455.

6. Asbun HJ, Rossi RL, Lowell JA, Munson JL. Bile duct injury during laparoscopic cholecystectomy: mechanism of injury, prevention, and management. *World J Surg.*17(4):547-551; 551-542.

7. Bachellier P, Nakano H, Weber JC, et al. Surgical repair after bile duct and vascular injuries during laparoscopic cholecystectomy: when and how? *World J Surg.*25(10):1335-1345.

8. Bakhsh R, Zahid MA, Dar FS, Malik ZI, Akhtar N, Akhtar S. Iatrogenic bile duct injuries: experience at PIMS. *J Ayub Med Coll Abbottabad.*14(4):16-18.

9. Bansal VK, Krishna A, Misra MC, et al. Factors Affecting Short-Term and Long-Term Outcomes After Bilioenteric Reconstruction for Post-cholecystectomy Bile Duct Injury: Experience at a Tertiary Care Centre. *Indian J Surg.*77:472-479.

10. Barros F, Fern, es RA, de Oliveira ME, Pacheco LF, Martinho JM. The influence of time referral in the treatment of iatrogenic lesions of biliary tract. *Rev Col Bras Cir.*37(6):407-412.

11. Barrow PJ, Siriwardena AK. Outcome of hepaticojejunostomy without access loop for repair of iatrogenic bile duct injury at laparoscopic cholecystectomy. *J Hepatobiliary Pancreat Surg.* 2007;14(4):374-376.

12. Bauer TW, Morris JB, Lowenstein A, Wolferth C, Rosato FE, Rosato EF. The consequences of a major bile duct injury during laparoscopic cholecystectomy. *J Gastrointest Surg.*2(1):61-66.

13. Bektas H, Schrem H, Winny M, Klempnauer J. Surgical treatment and outcome of iatrogenic bile duct lesions after cholecystectomy and the impact of different clinical classification systems. *Br J Surg.*94(9):1119-1127.

14. Bingham J, McKie LD, McLoughlin J, Diamond T. Biliary complications associated with laparoscopic cholecystectomy: analysis of common misconceptions. *Br J Surg.*87(3):362-373.

15. Bobkiewicz A, Krokowicz L, Banasiewicz T, et al. Iatrogenic bile duct injury. A significant surgical problem. Assessment of treatment outcomes in the department's own material. *Pol Przegl Chir.*86(12):576-583.

16. Boerma D, Rauws EA, Keulemans YC, et al. Impaired quality of life 5 years after bile duct injury during laparoscopic cholecystectomy: a prospective analysis. *Ann Surg.*234(6):750-757.

17. Branum G, Schmitt C, Baillie J, et al. Management of major biliary complications after laparoscopic cholecystectomy. *Ann Surg.*217(5):532-540; discussion 540-531.

18. Browder IW, Dowling JB, Koontz KK, Litwin MS. Early management of operative injuries of the extrahepatic biliary tract. *Ann Surg.*205(6):649-658.

19. Buell JF, Cronin DC, Funaki B, et al. Devastating and fatal complications associated with combined vascular and bile duct injuries during cholecystectomy. *Arch Surg.*137(6):703-708; discussion 708-710.

20. Cai ZF, Huang HL, Dui DH, Peng CJ, Zhao LJ. Early diagnosis and treatment of high bile duct damage caused by laparoscopic gallbladder excision. *World Chinese Journal of Digestology.* 2014(22):3316-3319.

21. Chaudhary A, Ch, ra A, Negi SS, Sachdev A. Reoperative surgery for postcholecystectomy bile duct injuries. *Dig Surg.* 2002;19(1):22-27.

22. Cie?licki J, Drews M, Marciniak R, Ratajczak A, Stajgis P, Dryjas A. Reconstructive surgery of bile ducts from personal material. *Wiadomosci lekarskie (Warsaw, Poland*1997:323-325.

23. Csendes A, Diaz JC, Burdiles P, Maluenda F. Late results of immediate primary end to end repair in accidental section of the common bile duct. *Surg Gynecol Obstet.*168(2):125-130.

24. Cuendis-Velazquez A, Bada-Yllan O, Trejo-Avila M, et al. Robotic-assisted Roux-en-Y hepaticojejunostomy after bile duct injury. *Langenbecks Arch Surg.*403(1):53-59.

25. Cuendis-Velazquez A, Morales-Chavez C, Aguirre-Olmedo I, et al. Laparoscopic hepaticojejunostomy after bile duct injury. *Surg Endosc.*30(3):876-882.

26. Dageforde LA, L., man MP, et al. A cost-effectiveness analysis of early vs late reconstruction of iatrogenic bile duct injuries. *J Am Coll Surg.*214(6):919-927.

27. de Reuver PR, Grossmann I, Busch OR, Obertop H, van Gulik TM, Gouma DJ. Referral pattern and timing of repair are risk factors for complications after reconstructive surgery for bile duct injury. *Ann Surg.*245(5):763-770.

28. de S V, e S, Bossens M, Parmentier Y, Gigot JF. National survey on cholecystectomy related bile duct injury--public health and financial aspects in Belgian hospitals--1997. *Acta Chir Belg.*103(2):168-180.

29. de Santibanes E, Palavecino M, Ardiles V, Pekolj J. Bile duct injuries: management of late complications. *Surg Endosc.*20(11):1648-1653.

30. Efskind L, Helsingen Jr N. Operative lesions of the bile ducts. *Acta Chirurgica Scandinavica.* 1956;111(6):475-480.

31. Elhamel A, Nagmuish S, Elfaidi S, Ben Dalal H. Handling of biliary complications following laparoscopic cholecystectomy in the setting of Tripoli Central Hospital. *HPB (Oxford).* 2008;4(3):105-110.

32. Fathy O, Wahab MA, Hamdy E, et al. Post-cholecystectomy biliary injuries: one center experience. *Hepatogastroenterology.*58(107):719-724.

33. Fischer CP, Fahy BN, Aloia TA, Bass BL, Gaber AO, Ghobrial RM. Timing of referral impacts surgical outcomes in patients undergoing repair of bile duct injuries. *HPB (Oxford).*11(1):32-37.

34. Frilling A, Li J, Weber F, et al. Major bile duct injuries after laparoscopic cholecystectomy: a tertiary center experience. *J Gastrointest Surg.*8(6):679-685.

35. Gazzaniga GM, Filauro M, Mori L. Surgical treatment of iatrogenic lesions of the proximal common bile duct. *World J Surg.*25(10):1254-1259.

36. Giulianotti PC, Quadri P, Durgam S, Bianco FM. Reconstruction/Repair of Iatrogenic Biliary Injuries: Is the Robot Offering a New Option? Short Clinical Report. *Ann Surg.*267(1):e7-e9.

37. Gouma DJ, Go PM. Bile duct injury during laparoscopic and conventional cholecystectomy. *J Am Coll Surg.*178(3):229-233.

38. Gouma DJ, Rauws EA, Keulemans YC, Bergman JJ, Huibregtse K, Obertop H. [Bile duct injuries after a laparoscopic cholecystectomy]. *Ned Tijdschr Geneeskd.*143(12):606-611.

39. Gupta RK, Agrawal CS, Sah S, Sapkota S, Pathania OP, Sah PL. Bile duct injuries during open and laparoscopic cholecystectomy: management and outcome. *J Nepal Health Res Counc.*11(24):187-193.

40. Hadi A, Aman Z, Khan SA, et al. Surgical management of bile duct injuries following open or laparoscopic cholecystectomy. *Journal of the Pakistan Medical Association.*63(8):1008-1012.

41. Hajjar NA, Tomus C, Mocan L, et al. Management of bile duct injuries following laparoscopic cholecystectomy: long-term outcome and risk factors infuencing biliary reconstruction. *Chirurgia (Bucur).*109(4):493-499.

42. Halbert C, Altieri MS, Yang J, et al. Long-term outcomes of patients with common bile duct injury following laparoscopic cholecystectomy. *Surg Endosc.*30(10):4294-4299.

43. Hart RS, Passi RB, Wall WJ. Long-term outcome after repair of major bile duct injury created during laparoscopic cholecystectomy. *Hpb.* 2000;2(3):325-332.

44. Helmy A, Gad H, Hammad E, et al. Iatrogenic biliary injuries: Patterns and surgical management. *Digestive Surgery.* 1997;14(6):534-539.

45. Hofmeyr S, Krige JE, Bornman PC, Beningfield SJ. A cost analysis of operative repair of major laparoscopic bile duct injuries. *S Afr Med J.*105(6):454-457.

46. Hogan NM, Dorcaratto D, Hogan AM, et al. Iatrogenic common bile duct injuries: Increasing complexity in the laparoscopic era: A prospective cohort study. *Int J Surg.*33:151-156.

47. Holte K, Bardram L, Wettergren A, Rasmussen A. Reconstruction of major bile duct injuries after laparoscopic cholecystectomy. *Dan Med Bull.*57(2):A4135.

48. Huang Q, Liuz CH, Zhu CL, Xiez F, Hu SY. The choice of surgical timing for biliary duct reconstruction after obstructive bile duct injury: an experimental study. *Hepatogastroenterology.*60(128):1865-1872.

49. Huang Q, Shao F, Qiu LJ, Wang C. Early vs. delayed repair of isolated segmental, sectoral and right hepatic bile duct injuries. *Hepatogastroenterology.*58(107):725-728.

50. Jablonska B, Lampe P, Olakowski M, Gorka Z, Lekstan A, Gruszka T. Hepaticojejunostomy vs. end-to-end biliary reconstructions in the treatment of iatrogenic bile duct injuries. *J Gastrointest Surg.*13(6):1084-1093.

51. Jackson N, Dugan A, Davenport D, et al. Risk factors for increased resource utilization and critical care complications in patients undergoing hepaticojejunostomy for biliary injuries. *HPB (Oxford).*18(9):712-717.

52. Jayasundara JA, de Silva WM, Pathirana AA. Changing clinical profile, management strategies and outcome of patients with biliary tract injuries at a tertiary care center in Sri Lanka. *Hepatobiliary Pancreat Dis Int.*10(5):526-532.

53. Johnson SR, Koehler A, Pennington LK, Hanto DW. Long-term results of surgical repair of bile duct injuries following laparoscopic cholecystectomy. *Surgery.*128(4):668-677.

54. Joshi RM, Shetty TS, Singh R, Adhikari DR, Patil BP, Bhange SA. Biliary complications of cholecystectomy. *Int Surg.*93(5):251-256.

55. Kaman L, Sanyal S, Behera A, Singh R, Katariya RN. Comparison of major bile duct injuries following laparoscopic cholecystectomy and open cholecystectomy. *ANZ J Surg.*76(9):788-791.

56. Karabulut M, Bas K, Gonenc M, et al. Diagnosis and treatment of iatrogenic bile duct injuries. *Medical Journal of Bakirkoy.* 2012;8(3):116-122.

57. Karvonen J, Gullichsen R, Laine S, Salminen P, Gronroos JM. Bile duct injuries during laparoscopic cholecystectomy: primary and long-term results from a single institution. *Surg Endosc.*21(7):1069-1073.

58. Kazibudzki M, Orawczyk T, Urbanek T, et al. Iatrogenic trauma of the biliary tract - Own experience. [Polish, English]. *Chirurgia Polska.* 2006;8(4):243-251.

59. Kerimoglu RS, Bostanci EB, Dalgic T, et al. Management of Laparoscopic Cholecystectomy-Related Bile Duct Injuries: A Tertiary Center Experience. *Arch Iran Med.*20(8):487-493.

60. Keulemans YC, Bergman JJ, de Wit LT, et al. Improvement in the management of bile duct injuries? *J Am Coll Surg.*187(3):246-254.

61. Krahenbuhl L, Sclabas G, Wente MN, Schafer M, Schlumpf R, Buchler MW. Incidence, risk factors, and prevention of biliary tract injuries during laparoscopic cholecystectomy in Switzerland. *World J Surg.*25(10):1325-1330.

62. Kune GA. Bile duct injury during cholecystectomy: causes, prevention and surgical repair in 1979. *Aust N Z J Surg.*49(1):35-40.

63. Li LB, Cai XJ, Mou YP, Wei Q, Wang XF. Factors influencing the results of treatment of bile duct injuries during laparoscopic cholecystectomy. *Hepatobiliary Pancreat Dis Int.*4(1):113-116.

64. Lillemoe KD, Martin SA, Cameron JL, et al. Major bile duct injuries during laparoscopic cholecystectomy. Follow-up after combined surgical and radiologic management. *Ann Surg.*225(5):459-468; discussion 468-471.

65. Lillemoe KD, Melton GB, Cameron JL, et al. Postoperative bile duct strictures: management and outcome in the 1990s. *Ann Surg.*232(3):430-441.

66. Linhares BL, Magalhaes Ada G, Cardoso PM, Linhares Filho JP, Pinho JE, Costa ML. Bile duct injury following cholecystectomy. *Rev Col Bras Cir.*38(2):95-99.

67. Lubikowski J, Post M, Bialek A, Kordowski J, Milkiewicz P, Wojcicki M. Surgical management and outcome of bile duct injuries following cholecystectomy: a single-center experience. *Langenbecks Arch Surg.*396(5):699-707.

68. Ludwig K, Bernhardt J, Steffen H, Lorenz D. Contribution of intraoperative cholangiography to incidence and outcome of common bile duct injuries during laparoscopic cholecystectomy. *Surg Endosc.*16(7):1098-1104.

69. Maddah G, Rajabi Mashhadi MT, Parvizi Mashhadi M, Nooghabi MJ, Hassanpour M, Abdollahi A. Iatrogenic injuries of the extrahepatic biliary system. *J Surg Res.*213:215-221.

70. Martinez-Lopez S, Upasani V, P., et al. Delayed referral to specialist centre increases morbidity in patients with bile duct injury (BDI) after laparoscopic cholecystectomy (LC). *International Journal of Surgery.*44:82-86.

71. Mathisen O, Soreide O, Bergan A. Laparoscopic cholecystectomy: bile duct and vascular injuries: management and outcome. *Scand J Gastroenterol.*37(4):476-481.

72. Mercado MA, Chan C, Orozco H, Tielve M, Hinojosa CA. Acute bile duct injury. The need for a high repair. *Surg Endosc.*17(9):1351-1355.

73. Mercado MA, Chan C, Salgado-Nesme N, Lopez-Rosales F. Intrahepatic repair of bile duct injuries. A comparative study. *J Gastrointest Surg.*12(2):364-368.

74. Mercado MA, Franssen B, Dominguez I, et al. Transition from a low: to a high-volume centre for bile duct repair: changes in technique and improved outcome. *HPB (Oxford).*13(11):767-773.

75. Mihaileanu F, Zaharie F, Mocan L, Iancu C, Vlad L. Management of bile duct injuries secondary to laparoscopic and open cholecystectomy. The experience of a single surgical department. *Chirurgia (Bucur).*107(4):454-460.

76. Mishra PK, Saluja SS, Nayeem M, Sharma BC, Patil N. Bile Duct Injury-from Injury to Repair: an Analysis of Management and Outcome. *Indian J Surg.*77:536-542.

77. Moossa AR, Mayer AD, Stabile B. Iatrogenic injury to the bile duct. Who, how, where? *Arch Surg.*125(8):1028-1030; discussion 1030-1021.

78. Moraca RJ, Lee FT, Ryan JA, Jr., Traverso LW. Long-term biliary function after reconstruction of major bile duct injuries with hepaticoduodenostomy or hepaticojejunostomy. *Arch Surg.*137(8):889-893; discussion 893-884.

79. Nordin A, Halme L, Makisalo H, Isoniemi H, Hockerstedt K. Management and outcome of major bile duct injuries after laparoscopic cholecystectomy: from therapeutic endoscopy to liver transplantation. *Liver Transpl.*8(11):1036-1043.

80. Nuzzo G, Giuliante F, Giovannini I. Timing of repair of bile duct injuries associated with laparoscopic cholecystectomy. *Arch Surg.*146(1):117; author reply 117-117; author reply 118.

81. Nuzzo G, Giuliante F, Giovannini I, et al. Advantages of multidisciplinary management of bile duct injuries occurring during cholecystectomy. *Am J Surg.*195(6):763-769.

82. Ozturk E, Can MF, Yagci G, et al. Management and mid- to long-term results of early referred bile duct injuries during laparoscopic cholecystectomy. *Hepatogastroenterology.*56(89):17-25.

83. Parks RW, Spencer EFA, McIlrath EM, Johnston GW. Review of the management of iatrogenic bile duct injuries. *Irish Journal of Medical Science.* 1994;163(12):571-575.

84. Patrono D, Benvenga R, Colli F, Baroffio P, Romagnoli R, Salizzoni M. Surgical management of post-cholecystectomy bile duct injuries: referral patterns and factors influencing early and long-term outcome. *Updates Surg.*67(3):283-291.

85. Pekolj J, Alvarez FA, Palavecino M, Sanchez Claria R, Mazza O, de Santibanes E. Intraoperative management and repair of bile duct injuries sustained during 10,123 laparoscopic cholecystectomies in a high-volume referral center. *J Am Coll Surg.*216(5):894-901.

86. Pottakkat B, Sikora SS, Kumar A, Saxena R, Kapoor VK. Recurrent bile duct stricture: causes and long-term results of surgical management. *J Hepatobiliary Pancreat Surg.* 2007;14(2):171-176.

87. Pulitan C, Parks RW. Current management of iatrogenic bile duct injuries. *Surgery.*28(5):222-225.

88. Quintero GA, Patino JF. Surgical management of benign strictures of the biliary tract. *World J Surg.*25(10):1245-1250.

89. Raute M, Podlech P, Jaschke W, Manegold BC, Trede M, Chir B. Management of bile duct injuries and strictures following cholecystectomy. *World J Surg.*17(4):553-562.

90. Ress AM, Sarr MG, Nagorney DM, Farnell MB, Donohue JH, McIlrath DC. Spectrum and management of major complications of laparoscopic cholecystectomy. *Am J Surg.*165(6):655-662.

91. Richardson MC, Bell G, Fullarton GM. Incidence and nature of bile duct injuries following laparoscopic cholecystectomy: an audit of 5913 cases. West of Scotland Laparoscopic Cholecystectomy Audit Group. *Br J Surg.*83(10):1356-1360.

92. Riggs T, Foshag L, Vargish T, Zimmerman B. Biliary tract injuries following routine cholecystectomy. *American Surgeon.* 1986;52(6):312-314.

93. Rosenqvist H, Myrin SO. Operative injuries to the bile ducts. *Acta Chirurgica Scandinavica.* 1960;119(2):92-107.

94. Rossi RL, Schirmer WJ, Braasch JW, S., ers LB, Munson JL. Laparoscopic bile duct injuries. Risk factors, recognition, and repair. *Arch Surg.*127(5):596-601; discussion 601-592.

95. Roy AF, Passi RB, Lapointe RW, McAlister VC, Dagenais MH, Wall WJ. Bile duct injury during laparoscopic cholecystectomy. *Canadian Journal of Surgery.* 1993;36(6):509-516.

96. Russell JC, Walsh SJ, Mattie AS, Lynch JT. Bile duct injuries, 1989-1993. A statewide experience. Connecticut Laparoscopic Cholecystectomy Registry. *Arch Surg.*131(4):382-388.

97. Rystedt J, Lindell G, Montgomery A. Bile Duct Injuries Associated With 55,134 Cholecystectomies: Treatment and Outcome from a National Perspective. *World J Surg.*40(1):73-80.

98. Saber K, Manialawi M. Repair of bile duct injuries. *World Journal of Surgery.* 1984;8(1):82-89.

99. Sadegh Fazeli M, Kazemeini AR, Jafarian A, Bashashati M, Keramati MR. Temporary Trans-jejunal Hepatic Duct Stenting in Roux-en-y Hepaticojejunostomy for Reconstruction of Iatrogenic Bile Duct Injuries. *Trauma Mon.*21(2):e21115.

100. Salama IA, Shoreem HA, Saleh SM, et al. Iatrogenic biliary injuries: multidisciplinary management in a major tertiary referral center. *HPB Surg.* 2014;2014:575136.

101. Sanei B, Aria A, Jafari HR. Evaluating surgery outcomes in patients with bile duct injury following cholecystectomy according to referral pattern; a five-year study. *Journal of Isfahan Medical School.*35(461):1867-1872.

102. Savar A, Carmody I, Hiatt JR, Busuttil RW. Laparoscopic bile duct injuries: management at a tertiary liver center. *Am Surg.*70(10):906-909.

103. Sawaya DE, Jr., Johnson LW, Sittig K, McDonald JC, Zibari GB. Iatrogenic and noniatrogenic extrahepatic biliary tract injuries: a multi-institutional review. *Am Surg.*67(5):473-477.

104. Schmidt SC, Settmacher U, Langrehr JM, Neuhaus P. Management and outcome of patients with combined bile duct and hepatic arterial injuries after laparoscopic cholecystectomy. *Surgery.*135(6):613-618.

105. Schol FP, Go PM, Gouma DJ. Outcome of 49 repairs of bile duct injuries after laparoscopic cholecystectomy. *World J Surg.*19(5):753-756; discussion 756-757.

106. Seeliger H, Furst A, Zulke C, Jauch KW. Surgical management of bile duct injuries following laparoscopic cholecystectomy: analysis and follow-up of 28 cases. *Langenbecks Arch Surg.*387(7):286-293.

107. Shah SR, Mirza DF, Afonso R, Mayer AD, McMaster P, Buckels JA. Changing referral pattern of biliary injuries sustained during laparoscopic cholecystectomy. *Br J Surg.*87(7):890-891.

108. Shaikh R, Pohani MR, Ayub, Asghar A, Malik KA, Ur Rehman S. Bile duct injuries during open and laproscopic cholecystectomy: Management and outcome. *Pakistan Journal of Medical Sciences.*25(3):496-499.

109. Sicklick JK, Camp MS, Lillemoe KD, et al. Surgical management of bile duct injuries sustained during laparoscopic cholecystectomy: perioperative results in 200 patients. *Ann Surg.*241(5):786-792; discussion 793-785.

110. Sikora SS, Kumar A, Das NR, Sarkari A, Saxena R, Kapoor VK. Laparoscopic bile duct injuries: spectrum at a tertiary-care center. *J Laparoendosc Adv Surg Tech A.*11(2):63-68.

111. Sikora SS, Pottakkat B, Srikanth G, Kumar A, Saxena R, Kapoor VK. Postcholecystectomy benign biliary strictures - long-term results. *Dig Surg.* 2006;23(5):304-312.

112. Slater K, Strong RW, Wall DR, Lynch SV. Iatrogenic bile duct injury: the scourge of laparoscopic cholecystectomy. *ANZ J Surg.*72(2):83-88.

113. Strasberg SM, Picus DD, Drebin JA. Results of a new strategy for reconstruction of biliary injuries having an isolated right-sided component. *J Gastrointest Surg.*5(3):266-274.

114. Sulpice L, Garnier S, Rayar M, Meunier B, Boudjema K. Biliary cirrhosis and sepsis are two risk factors of failure after surgical repair of major bile duct injury post-laparoscopic cholecystectomy. *Langenbecks Arch Surg.*399(5):601-608.

115. Thomson BN, Cullinan MJ, Banting SW, Collier NA. Recognition and management of biliary complications after laparoscopic cholecystectomy. *ANZ J Surg.*73(4):183-188.

116. Topal B, Aerts R, Penninckx F. The outcome of major biliary tract injury with leakage in laparoscopic cholecystectomy. *Surgical Endoscopy.*13(1):53-56.

117. Tropea A, Pagano D, Biondi A, Spada M, Gruttadauria S. Treatment of the iatrogenic lesion of the biliary tree secondary to laparoscopic cholecystectomy: a single center experience. *Updates Surg.*68(2):143-148.

118. Tsalis KG, Christoforidis EC, Dimitriadis CA, Kalfadis SC, Botsios DS, Dadoukis JD. Management of bile duct injury during and after laparoscopic cholecystectomy. *Surg Endosc.*17(1):31-37.

119. Viikari SJ. Operative injuries to the bile ducts. Experience of 49 cases. *Acta Chirurgica Scandinavica.* 1960;119(2):83-92.

120. Viste A, Horn A, Ovrebo K, Christensen B, Angelsen JH, Hoem D. Bile duct injuries following laparoscopic cholecystectomy. *Scand J Surg.*104(4):233-237.

121. Walsh RM, Henderson JM, Vogt DP, et al. Trends in bile duct injuries from laparoscopic cholecystectomy. *J Gastrointest Surg.*2(5):458-462.

122. Wei AC. Timing of repair of bile duct injuries associated with laparoscopic cholecystectomy: In reply. *Archives of Surgery.*146(1):117-118.

123. Woods MS, Traverso LW, Kozarek RA, et al. Characteristics of biliary tract complications during laparoscopic cholecystectomy: a multi-institutional study. *Am J Surg.*167(1):27-33; discussion 33-24.

124. Wu JS, Peng C, Mao XH, Lv P. Bile duct injuries associated with laparoscopic and open cholecystectomy: sixteen-year experience. *World J Gastroenterol.*13(16):2374-2378.

125. Wudel LJ, Jr., Wright JK, Pinson CW, et al. Bile duct injury following laparoscopic cholecystectomy: a cause for continued concern. *Am Surg.*67(6):557-563; discussion 563-554.

126. Xu XD, Zhang YC, Gao P, et al. Treatment of major laparoscopic bile duct injury: a long-term follow-up result. *Am Surg.*77(12):1584-1588.

127. Yeh TS, Jan YY, Wang CS, Jeng LB, Hwang TL, Chen MF. A multidisciplinary approach to major bile duct injury following laparoscopic cholecystectomy. *Jsls.*2(2):147-151.

| **Table S2** Newcastle–Ottawa risk-of-bias table | | | | | | | | | |
| --- | --- | --- | --- | --- | --- | --- | --- | --- | --- |
| Study | NOS score | S1. | S2. | S3. | S4. | Q1. | O1. | O2. | O3. |
| Ooi | 5 | A* | A* | A* | A* | 0 | B* | B | D |
| Thomson | 6 | B* | A* | A* | A* | * | B* | B | D |
| Akaraviputh | 5 | A* | A* | A* | A* | 0 | B* | B | D |
| Walsh | 8 | A* | A* | A* | A* | ** | B* | A* | D |
| Goykhman | 6 | A* | A* | A* | A* | * | B* | B | D |
| Stewart | 7 | A* | A* | A* | A* | ** | B* | B | D |
| Winslow | 7 | A* | A* | A* | A* | * | B* | B | A* |
| Sahajpal | 7 | B* | A* | A* | A* | * | B* | A* | D |
| Parera | 6 | B* | A* | A* | A* | * | B* | B | D |
| Ianelli | 5 | B* | A* | A* | A* | 0 | B* | B | D |
| Pitt | 6 | A* | B | A* | A* | * | B* | B | B* |
| Gluszek | 5 | A* | A* | A* | A* | 0 | B* | B | D |
| Huang | 6 | A* | A* | A* | A* | 0 | B* | A* | D |
| Stilling | 6 | A* | A* | A* | A* | 0 | B* | A* | D |
| Felekouras | 7 | A* | A* | A* | A* | 0 | B* | A* | A* |
| Gomes | 8 | A* | A* | A* | A* | * | B* | A* | A* |
| Rystedt | 6 | A* | A* | A* | A* | 0 | B* | B | B |
| Dominguez-Rosado | 7 | B* | A* | A* | A* | ** | B* | B | D |
| Kirks | 7 | B* | A* | A* | A* | ** | B* | B | D |
| Ismael | 6 | B* | A* | A* | A* | 0 | B* | B | A* |
| Booij | 7 | A* | B | A* | A* | ** | B* | A* | D |
| * = 1 point | | | | | | | | | |
| **Selection** | | | | | | | | | |
| S1. Representativeness of exposed cohort | | | | | | | | | |
| S2. Selection of the non-exposed cohort | | | | | | | | | |
| S3. Ascertainment of exposure | | | | | | | | | |
| S4. Demonstration that outcome of interest was not present at start of study | | | | | | | | | |
| **Comparability** | | | | | | | | | |
| C1. Comparability of cohorts on the basis of the design or analysis | | | | | | | | | |
| **Outcome** | | | | | | | | | |
| O1. Assessment of outcome | | | | | | | | | |
| O2. Was follow-up long enough for outcomes to occur | | | | | | | | | |
| O3. Adequacy of follow up of cohorts | | | | | | | | | |

| **Table S3** Timing of surgical repair | | | | | | | | | | | | | |
| --- | --- | --- | --- | --- | --- | --- | --- | --- | --- | --- | --- | --- | --- |
| Study | N | Time (days) | | | | | | | | | | | |
|  |  | 0 days | 7 days 1 week | | | | 14 days 2 weeks | 21 days  3 weeks | 28 days  4 weeks | 42 days 6 weeks | 56 days 8 weeks | 90 days 3 months | 182 days 6 months |
| Ooi | 14 | 0 (n = 7) | > 1 day (n = 7) | | | | | | | | | | |
| Thomson | 47 | < 2 weeks (n = 25) | | | | | | 2 weeks – 6 months (n = 22) | | | | | |
| Akaraviputh | 19 | 0 (n = 5) | > 1 day (n = 14) | | | | | | | | | | |
| Walsh | 84 | < 7 days (n = 44) | | | | | > 7 days (n = 40) | | | | | | |
| Goykhman | 23 | 0 (n = 8) | 1-3 d (n = 5) | | | - | | | | | | > 8 weeks (n = 10) | |
| Stewart | 137 | 0-7 days  (n = 31) | | | | | 1-2 weeks  (n = 30) | 3-6 weeks  (n = 33) | | | > 6 weeks  (n = 43) | | |
| Winslow | 88 | < 6 weeks (n = 22) | | | | | | | | | > 6 weeks (n = 66) | | |
| Sahajpal | 69 | 0 - 72 hours (n = 13) | | | 72h - 6 weeks (n = 34) | | | | | | > 6 weeks (n = 22) | | |
| Parera | 112 | 0 (n = 28) | < 21 days (n = 43) | | | | | | > 21 days (n = 41) | | | | |
| Ianelli | 253 | 0 (n = 35) | < 45 days (n = 91) | | | | | | | | > 45 days (n = 127) | | |
| Pitt | 101 | < 2 weeks  (n = 20) | | | | | | 2-4 weeks  (n = 7) | | 4-6 weeks  (n = 12) | 6-8 weeks  (n = 5) | 8-24 weeks  (n = 16) | > 24 weeks (n = 41) |
| Gluszek* | 11 | 0 (n = 2) | < 45 days (n = 9) | | | | | | | | > 45 days (n = 0) | | |
| Huang | 94 | < 2 weeks (n = 52) | | | | | | > 2 weeks (n = 42) | | | | | |
| Stilling* | 139 | < 2 days (n = 49) | | 3 - 14 days (n = 66) | | | | > 14 days (n = 24) | | | | | |
| Felekouras | 56 | < 2 weeks  (n = 34) | | | | | | - | | | | | > 12 weeks  (n = 22) |
| Gomes | 40 | < 3 weeks (n = 20) | | | | | | | > 3 weeks (n = 20) | | | | |
| Rystedt | 30 | < 3 weeks (n = 21) | | | | | | | > 3 weeks (n = 9) | | | | |
| Dominguez-Rosado | 586 | < 7 days (n = 61) | | | | | 7 days - 6 weeks (n = 152) | | | | > 6 weeks (n = 374) | | |
| Kirks | 61 | < 48 hours  (n = 27) | > 48 hours  (n = 34) | | | | | | | | | | |
| Ismael | 239 | < 30 days (n = 100) | | | | | | | | > 30 days (n = 139) | | | |
| Booij* | 281 | < 14 days  (n = 19) | | | | | | 14 - 90 days  (n = 91) | | | | | > 90 days  (n = 171) |

**Fig. S1** Secondary outcomes

| Conclusion as provided by the studies | |
| --- | --- |
| - | No information regarding significance or conclusion provided |
| § | Non-significant trend favouring early reconstruction |
| ¥ | Significant in favour of early reconstruction |
| ¤ | Non-significant trend favouring delayed reconstruction |
| º | Significant in favour of delayed reconstruction |
| = | “No difference in outcomes” |

| **Need for re-intervention** | | | | | | | | | | | | | | | | | | | | | | | | | | | | | | | | | | | | | | | | |
| --- | --- | --- | --- | --- | --- | --- | --- | --- | --- | --- | --- | --- | --- | --- | --- | --- | --- | --- | --- | --- | --- | --- | --- | --- | --- | --- | --- | --- | --- | --- | --- | --- | --- | --- | --- | --- | --- | --- | --- | --- |
| Study | | N | | Time (days) | | | | | | | | | | | | | | | | | | | | | | | | | | | | | | | C | | | | | |
|  | |  | | 0 days | 7 days  1 week | | | | | 14 days 2 weeks | | | 21 3 weeks | | | 28 4 weeks | | 42 days 6 weeks | | | | | | | 56 days  2 months | | | | | 90 days 3 months | | | | 182 days 6 months |  | | | | |  |
| Thomson | | 47 | | 1/25 (4.0%) | | | | | | | | | 2/22 (9.1%) | | | | | | | | | | | | | | | | | | | | | | - | | | | | |
| Akaraviputh | | 19 | | 0/5 (0%) | 0/14 (0%) | | | | | | | | | | | | | | | | | | | | | | | | | | | | | | - | | | | | |
| Stilling | | 139 | | 5/49 (10.2%) | | | | | 6/66 (9.1%) | | | | 4/24 (16.7%) | | | | | | | | | | | | | | | | | | | | | | - | | | | | |
| Booij | | 281 | | 2/19 (10.5%) | | | | | | | | | 3/91 (3.3%) | | | | | | | | | | | | | | | | | | | | | 4/171 (2.3%) | = | | | | | |
| **Length of stay** | | | | | | | | | | | | | | | | | | | | | | | | | | | | | | | | | | | | | | |  |  |
| Study | | N | | Time (days) | | | | | | | | | | | | | | | | | | | | | | | | | | | | | | | C | | | |  |  |
|  | |  | | 0 days | 7 days  1 week | | | | | 14 days 2 weeks | | | 21 3 weeks | | | 28 4 weeks | | | 42 days 6 weeks | | | | | | 56 days  2 months | | | | | 90 days 3 months | | | | 182 days 6 months |  | | |  |  |  |
| Akaraviputh | | 19 | | 12.0.±3.94 | 22.29 ± 15.57 | | | | | | | | | | | | | | | | | | | | | | | | | | | | | | § | | | |  |  |
| Rystedt | | 30 | | Median 18 days (6-47) | | | | | | | | | | | | Median 70 days (33-132) | | | | | | | | | | | | | | | | | | | § | | | |  |  |
| Kirks | | 61 | | Median 7 (2-45) | | | | Median 8 (3-56) | | | | | | | | | | | | | | | | | | | | | | | | | | | = | | | |  |  |
| **Bile leakage** | | | | | | | | | | | | | | | | | | | | | | | | | | | | | | | | | | | | | | | |  |
| Study | | N | | Time (days) | | | | | | | | | | | | | | | | | | | | | | | | | | | | | | | | | C | | |  |
|  | |  | | 0 days | 7 days  1 week | | | | | 14 days 2 weeks | | | 21 3 weeks | | | 28 4 weeks | | 42 days 6 weeks | | | | 56 days  2 months | | | | | | | 90 days 3 months | | | | 182 days 6 months | | | |  |  |  |  |
| Stilling | | 139 | | 5/49.(10.2%) | | | | | 2/66 (3.0%) | | | | 0/24 (0%) | | | | | | | | | | | | | | | | | | | | | | | | - | | |  |
| Felekouras | | 56 | | 4/34 (11.8%) | | | | | | | | |  | | | | | | | | | | | | | | | | | | | | 3/22 (13.6%) | | | | = | | |  |
| Booij | | 281 | | 3/19 (15.8%) | | | | | | | | | 7/91 (7.7%) | | | | | | | | | | | | | | | | | | | | 8/171 (4.7%) | | | | = | | |  |
| **Intra-abdominal abscess** | | | | | | | | | | | | | | | | | | | | | | | | | | | | | | | | | | | | | | | |  |
| Study | | N | | Time (days) | | | | | | | | | | | | | | | | | | | | | | | | | | | | | | | | C | | | |  |
|  | |  | | 0 days | 7 days  1 week | | | | | 14 days 2 weeks | | | 21 3 weeks | | | 28 4 weeks | | 42 days 6 weeks | | | | | | 56 days  2 months | | | | 90 days 3 months | | | | 182 days 6 months | | | |  | |  |  |  |
| Stilling | | 139 | | 7/49 (14.3%) | | | | | 8/66 (12.1%) | | | | 5/24 (20.8%) | | | | | | | | | | | | | | | | | | | | | | | - | | | |  |
| **Haemorrhage** | | | | | | | | | | | | | | | | | | | | | | | | | | | | | | | | | | | | | | | |  |
| Study | | N | | Time (days) | | | | | | | | | | | | | | | | | | | | | | | | | | | | | | | | C | | | |  |
|  | |  | | 0 days | 7 days  1 week | | | | | 14 days 2 weeks | | | 21 3 weeks | | | 28 4 weeks | | | | 42 days 6 weeks | | | 56 days  2 months | | | | | 90 days 3 months | | | | 182 days 6 months | | | |  | |  |  |  |
| Stilling | | 139 | | 2/49 (4.1%) | | | | | 2/66 (3.0%) | | | | 3/24 (12.5%) | | | | | | | | | | | | | | | | | | | | | | | - | | | |  |
| **Recurrent cholangitis (long-term)** | | | | | | | | | | | | | | | | | | | | | | | | | | | | | | | | | | | | | | | |  |
| Study | | N | | Time (days) | | | | | | | | | | | | | | | | | | | | | | | | | | | | | | | C | | | | |  |
|  | |  | | 0 days | | | 7 days  1 week | | | | | 14 days 2 weeks | 21 3 weeks | | 28 4 weeks | | 42 days 6 weeks | | | | 56 days  2 months | | | | | | 90 days 3 months | | | | 182 days 6 months | | | |  | | |  |  |  |
| Ooi | | 14 | | 0/7.(0%) | | | 1/7 (14.3%) | | | | | | | | | | | | | | | | | | | | | | | | | | | | - | | | | |  |
| Akaraviputh | | 19 | | 0/5 (0%) | | | 0/14 (0%) | | | | | | | | | | | | | | | | | | | | | | | | | | | | - | | | | |  |
| Perera | | 112 | | 3/28.  (10.7%) | | | 5/43 (11.6%) | | | | | | | | 4/41 (9.8%) | | | | | | | | | | | | | | | | | | | | - | | | | |  |
| Stilling | | 139 | | 8/49 (16.3%) | | | | | 16/66 (24.2%) | | | | 8/24 (33.3%) | | | | | | | | | | | | | | | | | | | | | | - | | | | |  |
| Felekouras | | 56 | | 4/34 (11.8%) | | | | | | | | |  | | | | | | | | | | | | | | | | | | 3/22.(13.6%) | | | | = | | | | |  |
| **Stone formation (long-term)** | | | | | | | | | | | | | | | | | | | | | | | | | | | | | | | | | | | | | | | |  |
| Study | | N | | Time (days) | | | | | | | | | | | | | | | | | | | | | | | | | | | | | | | C | | | | |  |
|  | |  | | 0 days | | | 7 days  1 week | | | | | 14 days 2 weeks | 21 3 weeks | | 28 4 weeks | | 42 days 6 weeks | | | | 56 days  2 months | | | | | | 90 days 3 months | | | | 182 days 6 months | | | |  | | |  |  |  |
| Ooi | | 14 | | 0/7.(0%) | | | 1/7 (14.3%) | | | | | | | | | | | | | | | | | | | | | | | | | | | | - | | | | |  |
| **Long-term mortality (long-term)** | | | | | | | | | | | | | | | | | | | | | | | | | | | | | | | | | | | | | | | |  |
| Study | | N | | Time (days) | | | | | | | | | | | | | | | | | | | | | | | | | | | | | | | C | | | | |  |
|  | |  | | 0 days | | | 7 days  1 week | | | | | 14 days 2 weeks | 21 3 weeks | | 28 4 weeks | | 42 days 6 weeks | | | | 56 days  2 months | | | | | | 90 days 3 months | | | | 182 days 6 months | | | |  | | |  |  |  |
| Ooi | | 14 | | 0/7.(0%) | | | 0/7 (0%) | | | | | | | | | | | | | | | | | | | | | | | | | | | | - | | | | |  |
| Felekouras | | 56 | | 1/34 (2.9%) | | | | | | | | | | |  | | | | | | | | | | | | | | | | 1/22.(4.5%) | | | |  | | | | |  |
| **Redo hepaticojejunostomy (long-term)** | | | | | | | | | | | | | | | | | | | | | | | | | | | | | | | | | | | | | | | |  |
| Study | N | | Time (days) | | | | | | | | | | | | | | | | | | | | | | | | | | | | | | | | C | | | | |  |
|  |  | | 0 days | | | 7 days  1 week | | | | | 14 days 2 weeks | | 21 3 weeks | | 28 4 weeks | | 42 days 6 weeks | | | | 56 days  2 months | | | | | | 90 days 3 months | | | | 182 days 6 months | | | |  | | |  |  |  |
| Winslow | 88 | | 0/22 (0%) | | | | | | | | | | | | | | | | | | 0/66 (0%) | | | | | | | | | | | | | | - | | |  |  |  |
| Perera | 112 | | 1/28. (3.6%) | | | 1/43 (2.3%) | | | | | | | | | 2/41 (4.9%) | | | | | | | | | | | | | | | | | | | | - | | |  |  |  |
| Felekouras | 56 | | 0/34 (0%) | | | | | | | | | |  | | | | | | | | | | | | | | | | | | 0/22.(0%) | | | | - | | |  |  |  |
| **Long-term morbidity** | | | | | | | | | | | | | | | | | | | | | | | | | | | | | | | | | | | | | | | |  |
| Study | N | | Time (days) | | | | | | | | | | | | | | | | | | | | | | | | | | | | | | | | C | | | | |  |
|  |  | | 0 days | | | 7 days  1 week | | | | | 14 days 2 weeks | | 21 3 weeks | | 28 4 weeks | | 42 days 6 weeks | | | | 56 days  2 months | | | | | | 90 days 3 months | | | | 182 days 6 months | | | |  | | |  |  |  |
| Sahajpal | 69 | | 2/13 (15.4%) | | | | | | | | | | | 9/34 (26.5%) | | | | | | | | | | | | 0/22 (0%) | | | | | | | | | º | | |  |  |  |
| Perera | 112 | | 6/28 (21.4%) | | | 6/43 (14.0%) | | | | | | | | | 16/41 (39.0%) | | | | | | | | | | | | | | | | | | | | ¥ | | |  |  |  |
| Felekouras | 56 | | 8/34 (23.5%) | | | | | | | | | |  | | | | | | | | | | | | | | | | | | 7/22.(31.8%) | | | | = | | |  |  |  |
